# Data on Support Vector Machines (SVM) model to forecast photovoltaic power

**DOI:** 10.1016/j.dib.2016.08.024

**Published:** 2016-08-18

**Authors:** M. Malvoni, M.G. De Giorgi, P.M. Congedo

**Affiliations:** Department of Engineering for Innovation, University of Salento, 73100 Lecce, Italy

**Keywords:** Least Squares Support Vector Machines (LS-SVM), Forecast photovoltaic

## Abstract

The data concern the photovoltaic (PV) power, forecasted by a hybrid model that considers weather variations and applies a technique to reduce the input data size, as presented in the paper entitled “Photovoltaic forecast based on hybrid pca-lssvm using dimensionality reducted data” (M. Malvoni, M.G. De Giorgi, P.M. Congedo, 2015) [1]. The quadratic Renyi entropy criteria together with the principal component analysis (PCA) are applied to the Least Squares Support Vector Machines (LS-SVM) to predict the PV power in the day-ahead time frame. The data here shared represent the proposed approach results. Hourly PV power predictions for 1,3,6,12, 24 ahead hours and for different data reduction sizes are provided in Supplementary material.

**Specifications Table**

**Table:** 

Subject area	Engineer, Neurosciences, Physics.
More specific subject area	Renewable resource, Solar energy, Photovoltaic, Forecasting methods
Type of data	Table, Excel file
How data was acquired	Data were obtained implementing the proposed model in [1] through MATLAB code.
Data format	Processed
Experimental factors	Sensors for measurements of solar irradiance on plane of array, module temperature and ambient temperature are connected to a Supervisory Control And Data Acquisition (SCADA) system. Thought protocols Modbus, the measured parameters are available in the private web site. Input data are treated to referred them at same time step.
Experimental features	The pre-processed data of outdoor measurements and PV output power represent the input set to implement the forecasting models. LS-SVM in combination with technique to resize the training data is implemented to predict the PV power in ahead day time frame.
Data source location	Lecce, Italy (40° 19׳32"׳16 N, 18° 5׳52"׳44 E)
Data accessibility	Data are within this article

**Value of the data**

• The provided data may be useful to compare the PV system performance in different climatic conditions.
• The PV power predictions performed through LS-SVM, in combination with a technique to dimensionally reduce the training data, may be used to compare the prediction methods accuracy.
• The forecasted PV power can be applied in the planning, operation and management of power systems in view of the future development of smart grids.
• The prediction of PV power can be used to optimize the electric vehicles integration and to manage the recharging status [2].

## 1 Data

The hourly PV power predicted by LS-SVM, combined with a technique to reduce the size of the training data, is provided for different input data dimensions in Supplementary file (online). For each size, hourly PV power predictions are referred at 1,3,6,12, 24 ahead hours.

## 2 Experimental design, materials and methods

### 2.1 Experimental data

The shared data refer to a grid connected 960 kWp PV system sited in Lecce, Southern Italy (40°19’32’’16N, 18°5’52’’44E), to supply the electricity to the utilities of the University of Salento Campus. The 3000 monocrystalline silicon PV modules are installed on shelters as car parking roof. The PV modules present the same azimuth angle of 10°, but they are tilted of two different angle, 3° and 15° respectively. More technical details may be found in [3]. An integrated web-connected system is available to measure and monitor the weather parameters and the PV output power. The PV field is equipped of a pyranometer to measure of solar radiation on the module׳s plane every 1 minute. Temperature probes provide measurements of the ambient temperature and the module temperature every 10 min. PV output power is sampled every 1 min. The data as monitored are collected in web site with private access.

### 2.2 Data pre-processing

For more accurate predictions, the PV energy forecasting models request the knowledge of meteo parameters [4], [5]. Therefore the weather data, referred to one year of measurements from 06/03/2012 to 30/03/2013, was considered for a total number of 9.192 hourly samples. In order to align them to the same sample step of 1 h, a data pre-processing was performed. For this aim, hourly average values of temperatures, solar irradiance and PV power were computed, as defined in [6].

The same meteo data have already been applied by the authors in [7] to investigate a hybrid statistical models based on LS-SVM with Wavelet Decomposition (WD) and in [8] to propose a novel hybrid algorithm GLSSVM (Group Least Square Support Vector Machine), based on the combination of the LS-SVM and a unexplored neural network known as GMDH (Group Method of Data Handling) implementing different strategies (Direct, Recursive and DirRec) for multi-step ahead forecast.

### 2.3 Data processing method

In [1] a hybrid method based on an active selection of the support vectors, using the quadratic Renyi entropy criteria in combination with the PCA is proposed to forecast the PV power output at different ahead hours.

In order to implement the forecasting methods, the input dataset of the following parameters was considered:

• *S*, variable to represent the season in which the data was measured;
• *H*, hourly instant to refer the data;
• *T_a_*, ambient temperature (°C);
• *T*_mod_, module temperature (°C);
• *G*_3,*n*_ and *G*_15,*n*_ solar irradiance difference at tilt 3° and 15° respectively, defined as the difference between the solar irradiance on tilted plane and the related hourly clear sky irradiance for each hour *i*, and computed as follow:

(1) $$\begin{array}{cc} G_{k}\left(i\right)=\frac{I_{\mathrm{sky},k}\left(i\right)-I_{k}\left(i\right)}{I_{0}} & i=1,2\ldots 9.192\;and\;k=3{}^{\circ},15{}^{\circ} \end{array}$$

where *I_k_* is the measured irradiance for each plane of array, *I*_sky,*k*_ is the corresponded solar irradiance in clear sky condition and *I*_0_=1.000 W/m^2^ is the solar radiance at Standard Test Condition. Difference time series *G_k_*(*i*) was normalized as follows

(2) $$\begin{array}{cc} G_{k,n}\left(i\right)=\frac{G_{k}\left(i\right)}{{\mathrm{Max}}_{i=1}^{9.192}(G_{k}\left(i\right))} & i=1,2\ldots 9.192\;and\;\;k=3{}^{\circ},15{}^{\circ} \end{array}$$

• *T_h_*, target of PV output power at the *h* prediction time horizon given as the cumulated PV power in *h* consecutive hours and scaled to at its maximum value, defined by:

(3) $$\begin{array}{cc} T_{h}\left(i\right)=\frac{\sum_{i+1}^{i+h}P\left(k\right)}{{\mathrm{Max}}_{i}P\left(i\right)} & i=1,2\ldots 9.192\;and\;\text{h}=1,3,6,12,24 \end{array}$$

where *P*(*i*) is the hourly average values PV power as computed during the data pre-processing.

The hourly mean ambient temperature, module temperature, solar irradiance measured on two tilted planes (*I*_3_ and *I*_15_) and the hourly mean PV power are provided in [6]. The time series of *G*_*k*,*n*_ and *T_h_* were computed. The input dataset was divided in two subsets. The samples referred to May, August, November and February (6336 about the 35% of the total records) were applied to test and the remain months (2856, about 65% of records) to train them.

The outcomes of the forecasting models represent the cumulated and normalized power, predicted for each time horizon ${\hat{T}}_{h}\left(i\right)$. The output data referred to the train set and the test set are shared in Supplementary material, File 1. The time series ${\hat{T}}_{h}\left(i\right)$ is provided at five time horizons, implementing the proposed methodology for four cases, as summarized in Table 1. More details are provided in [1].

## Figures and Tables

**Table 1:** Implementation of LS-SVM model for different size of input data set.

	Description
LS-SVM 0	LS-SVM implemented without technique to dimensionally reduce the training data;
LS-SVM M1	LS-SVM implemented with technique to dimensionally reduce the training data to M1=192 records;
LS-SVM M2	LS-SVM implemented with technique to dimensionally reduce the training data to M2=672 records;
LS-SVM M3	LS-SVM implemented with technique to dimensionally reduce the training data to M3=1920 records;
